# International Sex Survey: Study protocol of a large, cross-cultural collaborative study in 45 countries

**DOI:** 10.1556/2006.2021.00063

**Published:** 2021-09-16

**Authors:** Beáta Bőthe, Mónika Koós, Léna Nagy, Shane W. Kraus, Marc N. Potenza, Zsolt Demetrovics

**Affiliations:** 1 Département de Psychologie, Université de Montréal, Montréal, Canada; 2 Institute of Psychology, ELTE Eötvös Loránd University, Budapest, Hungary; 3 Doctoral School of Psychology, ELTE Eötvös Loránd University, Budapest, Hungary; 4 Department of Psychology, University of Nevada, Las Vegas, Las Vegas, NV, USA; 5 Yale University School of Medicine, New Haven, CT, USA; 6 Connecticut Council on Problem Gambling, Wethersfield, CT, USA; 7 Connecticut Mental Health Center, New Haven, CT, USA; 8 Centre of Excellence in Responsible Gaming, University of Gibraltar, Gibraltar, Gibraltar

**Keywords:** addictive behavior, compulsive sexual behavior, impulsive behavior, pornography, sexuality, sexual well-being

## Abstract

**Background and aims:**

Limitations of research into sexuality and compulsive sexual behavior disorder (CSBD) include the use of simplistic methodological designs and the absence of quality and unified measurements, empirically supported theoretical models, and large, collaborative studies between laboratories. We aim to fill these gaps with the International Sex Survey (ISS, http://internationalsexsurvey.org/).

**Methods:**

The ISS is a large-scale, international, multi-lab, multi-language study using cross-sectional survey methods, involving more than 40 countries. Participants responding to advertisements complete a self-report, anonymous survey on a secure online platform. Collaborators from each country collect a community sample of adults with a minimum sample size of 2,000 participants with a gender ratio of approximately 50–50% men and women, including diverse individuals with respect to sexuality and gender. The ISS includes a wide range of sociodemographic questions and scales assessing a diverse set of sexual behaviors, pornography use, psychological characteristics, and potential comorbid disorders. Analyses are conducted within a structural equation modeling framework, including variable (e.g., measurement invariance tests) and person-centered approaches (e.g., latent profile analysis).

**Discussion and conclusions:**

The ISS will provide well-validated, publicly available screening tools, helping to eliminate significant measurement issues in the field of sexuality research and health care. It will provide important insights to improve the theoretical understanding of CSBD as well as help to identify empirically supported treatment targets for prevention and intervention programs. Following open-science practices and making study materials open-access, the ISS may serve as a blueprint for future large-scale research in addiction and sexuality research.

## Introduction

Sexuality encompasses basic yet complex behaviors and is an integral part of people’s well-being. Experiencing problems with one’s sexuality may cover a wide range of concerns (e.g., sexual dysfunctions or sexuality-related impulse control disorders) (World Health Organization, 2019), and may result in significant distress and functional impairment (Bőthe, Tóth-Király, Griffiths et al., 2021; Kraus et al., 2018). Compulsive sexual behavior disorder (CSBD; also known as sex addiction or hypersexual disorder/hypersexuality) has long been considered (Carnes, 1983) and appears prevalent in general populations (3–10% in national probability samples) (Grubbs et al., 2020). However, its systematic scientific examination has started to increase only a few decades ago and proliferated in the past 25 years (Grubbs et al., 2020; Kafka, 2010). As a result, CSBD, characterized by persistent patterns of failures to control intense sexual urges and behaviors that result in clinically significant distress and functional impairment, is now included in the 11^th^ revision of the *International Classification of Diseases* (ICD-11) (Kraus et al., 2018; World Health Organization, 2019). However, some crucial questions have yet to be addressed, as the field is still characterized mainly by simplistic methodological designs and often lacks quality and unified measurements, empirically supported theoretical models, and collaborative studies between laboratories (Grubbs et al., 2020; Grubbs & Kraus, 2021). We aim to fill these gaps with the International Sex Survey (ISS), which is a large, international, collaborative study using rigorous methods to examine sexual behaviors. Given its recent inclusion in ICD-11 and documented knowledge gaps, the ISS will focus on CSBD and one of its most prominent manifestations, problematic pornography use (PPU) (Reid et al., 2012; Wordecha et al., 2018). However, other sexual behaviors (e.g., paraphilias, risky sexual behaviors) are also included in the study, and similar knowledge gaps also exist for these behaviors.

The ISS’ first overarching aim is to provide publicly available screening tools that can reliably and validly assess a wide range of sexual behaviors. For example, one of the main issues in CSBD research is the lack of valid and unified measurement, resulting in difficulties in comparing findings across studies (Grubbs et al., 2020). Although the assessment of CSBD has started to converge as a result of the proposed hypersexual disorder criteria (Kafka, 2010) and those for ICD-11-defined CSBD (Kraus et al., 2018; World Health Organization, 2019), there is no gold standard assessment for CSBD. To address this issue, the Compulsive Sexual Behavior Disorder Scale was developed (CSBD-19) (Bőthe, Potenza, Griffiths, Kraus, et al., 2020), which is currently the only tool assessing CSBD based on the ICD-11 diagnostic guidelines. Although it was developed in an international manner (i.e., Germany, Hungary, and the US) and demonstrated strong psychometric properties, further examination is required to establish its psychometric properties (e.g., validity, reliability) in other populations, as it has only been tested in Western, educated, industrialized, rich, and democratic (WEIRD) countries (Klein, Savaş, & Conley, 2021).

As more than 80% of individuals with CSBD also report PPU (Reid et al., 2012; Wordecha et al., 2018), PPU may be the most prominent manifestation of CSBD, and the aforementioned measurement issues apply to PPU as well (Fernandez & Griffiths, 2021; Grubbs et al., 2020). Based on the recommendations of recent systematic literature reviews (Fernandez & Griffiths, 2021; Grubbs et al., 2020), the Problematic Pornography Consumption Scale (PPCS) (Bőthe, Tóth-Király, et al., 2018) and the Brief Pornography Screen (BPS) (Kraus et al., 2020) are the most psychometrically robust scales to assess PPU. Although these scales were validated in diverse populations (Bőthe, Lonza, Štulhofer, Demetrovics, 2020; Bőthe, Tóth-Király, Demetrovics, Orosz, 2021; Bőthe, Tóth-Király, et al., 2018; Chen et al., 2021; Kraus et al., 2020), further examination is needed to establish how these scales function in other populations. In sum, the ISS’ first aim not only consolidates the assessment of compulsive sexual behaviors, including PPU, as well as other sexual behaviors assessed in the study, which is an essential prerequisite for any future research, but also paves the way for the second study aim.

The ISS’ second overarching aim is to examine who may develop adaptive and maladaptive sexual behaviors and how they may do so. Prior work and recent calls have highlighted the need to better understand which populations may be at greater risk of developing CSBD and PPU, and which processes may underlie the development of CSBD and PPU (Bőthe, Tóth-Király, et al., 2019; Bőthe, Tóth-Király, Potenza, Orosz, & Demetrovics, 2020; Grubbs et al., 2020; Grubbs & Kraus, 2021; Kowalewska, Gola, Kraus, & Lew-starowicz, 2020; Kraus, Martino, & Potenza, 2016). As detailed elsewhere (Grubbs et al., 2020), most studies seem to approach CSBD and PPU without assumptions about their theoretical background and knowledge about factors contributing to the development of CSBD and PPU.

Currently, despite the inclusion of CSBD in ICD-11 (World Health Organization, 2019), there is not sufficient scientific evidence to conclusively determine whether CSBD and PPU should be considered as impulse control, compulsivity-related, or addictive disorders (Kor, Fogel, Reid, & Potenza, 2013; Kraus et al., 2016a, 2016b; Kraus et al., 2016; Potenza, Gola, Voon, Kor, & Kraus, 2017; Prause, Janssen, Georgiadis, Finn, & Pfaus, 2017; Sassover & Weinstein, 2020). Therefore, from a theoretical perspective, we will assess both impulsivity and compulsivity as transdiagnostic features to consider questions concerning the etiologies of CSBD and PPU (i.e., whether CSBD and PPU are rather characterized by impulsivity, compulsivity, or both, supporting the impulse control, compulsive, or addictive disorders models, respectively) (Bőthe et al., 2019; Fineberg et al., 2014).

In the case of PPU, two other relevant models have been introduced to the literature recently. The moral incongruence model of pornography use (Grubbs, Perry, Wilt, & Reid, 2019; Grubbs & Perry, 2019) posits that PPU may appear not only as a result of dysregulation (e.g., impulsivity), but also due to moral incongruence towards pornography use (i.e., feelings that one’s pornography use is not consistent with one’s beliefs, resulting in self-perceived PPU). Thus, dysregulated, high-frequency pornography use may not always be present in the case of PPU. Complementing this notion, the engagement model of (online) behaviors (Billieux, 2012; Billieux, Flayelle, Rumpf, & Stein, 2019; Bőthe, Tóth-Király, et al., 2020) proposes that high engagement in a given activity (i.e., high-frequency pornography use) may not always result in problematic engagement in behaviors (i.e., PPU). Thus, besides impulsivity and compulsivity, we will also examine PPU’s associations with moral incongruence towards pornography use and pornography use frequency, providing insights into the applicability of these models in cross-cultural settings as most previous research has been conducted in WEIRD countries. In addition, other relevant sociodemographic (e.g., sexual orientation, religiosity; Bőthe, Bartók, et al., 2018; Grubbs, Perry, et al., 2019), sexuality and romantic relationship-related (e.g., sexual function, sexual abuse; Bőthe et al., 2021; Slavin et al., 2020), and psychological characteristics (e.g., basic psychological needs; Bőthe, Tóth-Király, et al., 2020), and other psychiatric symptomatology (e.g., relating to depression, ADHD; Bőthe, Koós, Tóth-Király, Orosz, & Demetrovics, 2019; Grubbs, Volk, Exline, & Pargament, 2015) will also be assessed in the ISS. In sum, to make substantial advances in the upcoming years, rigorous and systematic examination of sociodemographic and psychological characteristics that may contribute to the development of CSBD and PPU need to be conducted using sophisticated methods.

The overarching aims of the ISS are to (1) provide publicly available screening tools that can reliably and validly assess different sexual behaviors, (2) identify populations at high risk of developing maladaptive sexual behaviors, and (3) study mechanisms underlying different sexual behaviors and identify potential risk and protective factors related to maladaptive sexual behaviors. In the ISS, we plan to use a theory-based approach with high-quality statistical methods and large multi-cultural and diverse samples (e.g., sex- and gender-diverse individuals).

## Methods

### Study design

The ISS (http://internationalsexsurvey.org/) is an international, multi-lab, multi-language study using cross-sectional, self-report survey methods. The principal and co-investigators planned the study protocol, prepared all materials (e.g., survey, guidelines for collaborators, study advertisement materials), invited collaborators, secured incentives (i.e., donation), and preregistered the study (https://osf.io/uyfra/?view_only=6e4f96b748be42d99363d58e32d511b8). They will manage all language versions of the survey and collected data, conduct the analyses, and write the first drafts of most subsequent manuscripts. Collaborators from each country will sign the collaboration agreement, translate the survey battery from English to the target language, following a pre-established translation protocol (Beaton, Bombardier, Guillemin, & Ferraz, 2000); pretest it in the target language; obtain ethical permission for the study in their country; and, collect an adult community sample in their country. Collaborators can co-author papers in a manner that is compatible with the prevailing standards. The list of participating countries is shown in Table 1.

**Table 1.** List of participating countries in the International Sex Survey

Africa	America	Asia	Europe	Oceania
Algeria	Bolivia	Bangladesh	Austria	Australia
Egypt	Brazil	China	Belgium	New Zealand
South Africa	Canada	India	Croatia	
	Colombia	Iran	Czech Republic	
	Ecuador	Iraq	France	
	Mexico	Israel	Germany	
	Panama	Japan	Gibraltar	
	Peru	Malaysia	Hungary	
	United States	Pakistan	Ireland	
		South Korea	Italy	
		Taiwan	Lithuania	
			North Macedonia	
			Poland	
			Portugal	
			Romania	
			Slovakia	
			Spain	
			Switzerland	
			Turkey	
			United Kingdom	

*Note*. The list includes the participating countries (i.e., who signed the collaboration agreement) as of July 1, 2021.

The project’s start date was February 2021, when collaborators were provided the complete study documentation. Data collection starts in October 2021 at the earliest. The estimated end date for data collection is December 2021 but may change depending on the success of recruitment. Collaborators will contact large, nationwide popular news websites in their countries to advertise the study, using standardized advertisement materials (e.g., templates of emails to contact news websites, study advertisement text, and study advertisement posters). The advertisement materials explicitly state that participation in the study is completely anonymous, and anyone meeting the eligibility criteria can participate in the study, promoting inclusivity and encouraging participants to share sensitive information. Collaborators may offer exclusive results to advertising partners (i.e., in exchange for advertisement, collaborators may write one or two paragraphs about basic descriptive information about the sample collected in their country and send it to the advertising partners after finishing the data collection). Based on previous data collection experiences (Bőthe, Potenza, et al., 2020; Bőthe et al., 2021), popular news websites are usually open to collaborating with academic researchers in exchange for exclusive results. Moreover, as an incentive for participation, we will inform participants that the principal and co-investigators would donate 50 cents (USD) to non-profit, sexuality-related international organizations (e.g., World Association for Sexual Health) for every completed survey, with a maximum of a 1000 USD donation. Participants will have the opportunity to select their preferred non-profit, sexuality-related international organization from a list after survey completion. For example, this data collection strategy worked well in previous data collections in Hungary and the US (Bőthe, Bartók, et al., 2018; Bőthe, Potenza, et al., 2020; Bőthe et al., 2021; Koós, Bőthe, et al., 2021; Kraus et al., 2016), resulting in large samples (*N* = 9,000–24,000), including individuals of all genders and sexual orientations and diverse socioeconomic characteristics, in a relatively short period of time (i.e., several weeks). As the ISS includes data on sensitive topics, we will not make all datasets publicly available. However, we may provide data upon request when permissible.

### Procedure and participants

Participants responding to the advertisements complete a self-report, anonymous survey on a secure online platform (Qualtrics Research Suite). Survey completion takes approximately 25–35 min. Those individuals are eligible to participate in the study who (a) are at least 18 years old (or the legal age to provide informed consent) and (b) understand any of the languages in which the survey is available. Those participants are excluded from the study who (a) fail two out of three attention questions (Thomas & Clifford, 2017), and/or (b) produce unengaged response patterns (e.g., contradictory answers to several questions). Participants are provided a country-specific list of mental health services if they feel the need to speak to someone or need help. As an incentive, we inform participants that we donate 50 cents to non-profit, sexuality-related international organizations (e.g., World Association for Sexual Health) for every completed survey, with a maximum of a $1,000 donation.

Collaborators from each country collect a community sample of adults with a minimum sample size of 2,000 participants, with a gender ratio of approximately 50-50% men and women, including sexual and sex/gender-diverse individuals. We chose a minimum of 2,000 participants from each country as we plan to examine specific sub-samples (e.g., based on sexual orientation). Moreover, we plan to conduct analyses that require large samples, such as person-centered statistical approaches. However, the field of person-centered analyses is still evolving when it comes to *a priori* sample size and power calculations. Compared to variable-centered analyses where well-established guidelines and methods are available (e.g., G*Power software), no formal person-centered guidelines have been established yet, and only some recommendations are available (Collins & Wugalter, 1992; Finch & Bronk, 2011; Gudicha, Tekle, & Vermunt, 2016). Thus, we chose a sample size sufficient for all analyses planned to be conducted without power issues.

### Measures

#### Sociodemographic characteristics

A wide range of sociodemographic questions is included in the ISS survey battery. Participants report their sex assigned at birth, gender identity (Bauer, Braimoh, Scheim, & Dharma, 2017), trans status, sexual orientation (Weinrich, 2014), acceptance of gender and sexual orientation (by the individual and the country in which they reside), age, highest level of education, current engagement in education, work status, place of residence, ethnic minority status, subjective socioeconomic status, relationship status, number of children, and religious affiliation. The complete list of variables and the order of presentation in the survey battery is shown in Table 2.

**Table 2.** Complete list of variables in the International Sex Survey in order of presentation for participants

Variable (Scale, Abbreviation)	Number of items
*Informed consent, country, and language choice*	
Country of residence	1
Informed consent	1
*Sociodemographic characteristics*	
Sex assigned at birth	1
Gender identity	1
Trans status	1
Sexual orientation	1
Acceptance of gender and sexual orientation (by the individual and the country they reside in)	4
Age	1
Highest level of education	1
Current education	1
Work status	1
Place of residence	1
Ethnic minority status	1
Subjective socioeconomic status	1
Relationship status	1
Children	1
Religious affiliation	1
Religiousness	3
*General questions about sexuality*	
Having any sexual experience with a partner	1
Age at first sexual experience with a partner	1
Total number of sexual partner (in and out of a relationship)	1
Past-year sexual frequency (in and out of a relationship)	1
Contraception use	1
Having any sexually transmitted infection	1
Treatment for sexually transmitted infection	1
Having ever masturbated	1
Past-year frequency of masturbation	1
*Romantic relationship*	
Length of the romantic relationship	1
Relationship satisfaction (Single-item Relationship Assessment Scale, RAS-1)	1
Past-year sexual frequency (with the partner)	1
Sexual satisfaction (Global Measure of Sexual Satisfaction, GMSEX)	5
*Sexuality-related measures*	
Having any casual sexual partners	1
Number of past-year casual sexual partners	1
Past-year casual sexual frequency	1
Compulsive sexual behavior (Compulsive Sexual Behavior Disorder Scale, CSBD-19)	19
Having ever sought treatment for compulsive sexual behaviors	1
Being currently under treatment for compulsive sexual behaviors	1
Sexual distress (Short version of the Sexual Distress Scale, SDS-3)	3
Risky sexual behaviors (Sexual Risk Survey, SRS)	1+23
Paraphilias (Paraphilic Disorders Scale, PDS)	1+22
Sexual desire (Sexual Desire Inventory-2, SDI-2)	14
Question on displaying the male-bodied vs. female-bodied sexual function measure	1
Sexual function (Arizona Sexual Experience Scale, ASEX)	5
Sexual assertiveness (Short version of the Sexual Assertiveness Questionnaire, SAQ)	9
Sexual abuse (Sexual Abuse History Questionnaire, SAHQ)	6
*Pornography use-related measures*	
Having ever used pornography	1
Age at first pornography use	1
Past-year frequency of pornography use	1
Time spent with pornography use per each session	1
Pornography use motivations (Pornography Use Motivations Scale, PUMS)	24
Problematic pornography use (Problematic Pornography Consumption Scale, PPCS; Brief Pornography Screen, BPS)	23
Self-perceived pornography addiction	1
Moral incongruence toward pornography use	1
Having ever sought treatment for pornography use	1
Being currently under treatment for pornography use	1
*Impulsivity, compulsivity, and basic psychological needs*	
Impulsivity (Short UPPS-P Impulsivity Scale, UPPS-P)	20
Compulsivity (Compulsive Personality Assessment Scale, CPAS)	8
Basic psychological needs (Basic Psychological Needs Satisfaction and Frustration Scale, BPNSFS)	24
*Psychiatric symptomatology and related characteristics*	
Depression and anxiety (Subsales of the Brief Symptom Inventory, BSI-18)	12
ADHD (Adult ADHD Self-Report Screening Scale, ASRS)	6
Alcohol use disorder (Alcohol Use Disorders Identification Test, AUDIT)	1+9
Substance use (Alcohol, Smoking and Substance Involvement Screening Test, ASSIST)	1+10
Gambling	2+2
Body mass index	2
Binge eating (Binge Eating Disorder Screener-7, BEDS-7)	1+6
Self-harm (P4 Suicidality Screener)	1+4
*General questions about physical and mental health*	
Mental illness or emotional problems, medication, side effects	1+3
Physical illness, medication, side effects	1+3
Other medication or treatment, side effects	1+2
Any sexuality-related problems, medication, side effects	1+2
Currently pregnant and weeks of pregnancy	1+1
Breastfeeding	1
*COVID-19*	
COVID-19 status	1
Being in a COVID-19 risk group	1
COVID-19-related social distancing	1
Being affected by COVID-19	1
COVID-19-related stress	1
*Attention testing questions*	
Attention testing question 1 – added to SDI-2 (item 3)	1
Attention testing question 2 – added to UPPS-P (item 17)	1
Attention testing question 3 – added to ASRS-5 (item 5)	1
**End of survey, list of resources for participants**	

*Note*. When the number of items appears in a format of “number” + “number”, it means that a screening item is included in the survey battery and only participants with a positive answer to the screening item receive the additional items.

#### General questions about mental and physical health

##### Mental and physical health

One item assesses the presence of any mental health and emotional problems. If participants report any mental health or emotional problems, they answer three additional questions concerning medication use and experiencing any general or sexuality-related side effects. One item assesses the presence of any physical health problems. If participants report any physical health problems, they answer three additional questions concerning medication use and experiencing any general or sexuality-related side effects.

##### Medication

One item assesses the use of medication for any other health issues or conditions. If participants report taking any medication, they answer two additional questions concerning experiencing any general and sexuality-related side effects.

##### Sexual problems

One item assesses the presence of any sexual problems. If participants report taking any medication, they answer two additional questions concerning medication use and experience any side effects of medication.

##### Pregnancy and breastfeeding

One item assesses whether female participants are pregnant at the time of data collection and how far they are in their pregnancy. One item assesses whether female participants are breastfeeding at the time of data collection.

##### COVID-19-related questions

Five items assess participants’ COVID-19-related characteristics, including infection status, belonging to a risk group, engagement in social distancing, extent of being affected by the pandemic, and levels of COVID-19-related stress.

#### Sexuality-related characteristics

##### General questions about sexuality

Before asking about any sexual experiences with a partner, we provide a definition of sexual experience for participants.^1^ Participants report if they had any sexual experience with a partner before, age at first sexual experience with a partner, total number of sexual partners in their life, past-year sexual frequency (in and out of a relationship), past-year sexual frequency with the romantic partner, having any casual sex partner in their lifetime, past-year sexual frequency with casual sexual partners, type of contraception use, presence of any sexually transmitted infection, treatment for any sexually transmitted infection, and past and present treatment-seeking for compulsive sexual behaviors. Length of current romantic relationship,^2^ lifetime masturbation, and past-year masturbation frequency are assessed. All general questions about sexuality are asked with one item and based on previous studies (Beáta Bőthe, Bartók, et al., 2018; Bőthe et al., 2021).

##### Relationship satisfaction

The Single-item Relationship Assessment Scale (RAS-1) (Fülöp et al., 2020) assesses participants’ relationship satisfaction levels (i.e., “In general, how satisfied are you with your relationship?”). Participants indicate their answers on a five-point scale (1=“I am not satisfied”; 5=“I am very satisfied”).

##### Sexual satisfaction

The five-item Global Measure of Sexual Satisfaction (GMSEX) (Lawrance & Byers, 1998) assesses partnered individuals’ level of sexual satisfaction. It includes five seven-point dimensions based on a semantic differential approach (i.e., very bad-very good, very unpleasant-very pleasant, very negative-very positive, very unsatisfying-very satisfying, worthless-valuable) answering the question of “Overall, how would you describe your sexual relationship with your partner?”.

##### Compulsive sexual behavior

The 19-item Compulsive Sexual Behavior Disorder Scale (CSBD-19) (Bőthe, Potenza, et al., 2020) assesses the extent of compulsive sexual urges, thoughts, and behaviors and their consequences in the past six months. The CSBD-19 includes five factors based on the ICD-11 diagnostic guidelines: control (three items, e.g., “Even though my sexual behavior was irresponsible or reckless, I found it difficult to stop.”), salience (three items, e.g., “Sex has been the most important thing in my life.”), relapse (three items, e.g., “I was not successful in reducing the amount of sex I had.”), dissatisfaction (three items, e.g., “I had sex even when I did not enjoy it anymore.”), and general and domain-specific negative consequences (seven items, e.g., “My sexual activities interfered with my work and/or education.”). Participants indicate their levels of agreement on a four-point scale (1=“totally disagree”; 4=“totally agree”). Before completing the scale, participants read a pre-established definition of sex.^3^

##### Sexual distress

The one-factor, three-item version of the Sexual Distress Scale (SDS) (Derogatis, Rosen, Leiblum, Burnett, & Heiman, 2002; Pâquet et al., 2018) assesses the frequency of experiencing distress due to sexual difficulties in the past month (e.g., “How often did you feel distressed about your sex life?”). Participants indicate their answers on a five-point scale (0=“never”; 4=“always”).

##### Risky sexual behaviors

Participants indicating engagement in any type of risky sexual behaviors (i.e., “yes” answer to a screening question) are presented the relevant items of the 23-item Sexual Risk Survey (SRS) (Turchik & Garske, 2009). The SRS assesses how many times participants engaged in different risky sexual behaviors in the past six months. The SRS includes items related to risky vaginal, oral, or anal sex (e.g., “How many times have you had vaginal intercourse without protection against pregnancy?”), sex with casual sexual partner(s) (e.g., “How many times have you had sex with someone you don’t know well or just met?”), using alcohol or drugs while engaging in sexual activities (e.g., “How many times have you or your partner used alcohol or drugs before or during sex?”), having sex with a partner before discussing sexual history (e.g., “How many times have you had sex with a new partner before discussing sexual history, IV drug use, disease status and other current sexual partners?”), having sex with someone who had many sexual partners (e.g., “How many times (that you know of) have you had sex with someone who has had many sexual partners?”), having sex with someone who had been sexually active before you were with them but had not been tested for sexually transmitted infections (e.g., “How many partners (that you know of) have you had sex with who had been sexually active before you were with them but had not been tested for STIs/HIV?”), having sex with someone the participant do not trust (e.g., “How many partners have you had sex with that you didn’t trust?”), and having sex with someone who was also engaging in sex with others during the same period (e.g., “How many times (that you know of) have you had sex with someone who was also engaging in sex with others during the same time period?”).

##### Paraphilic disorders

Participants indicating interest in any paraphilias (i.e., “yes” answer to a screening question) complete items related to the reported paraphilic interest. Paraphilic disorders are assessed based on the current ICD-11 guidelines (Koós et al., 2021). Exhibitionistic disorder, voyeuristic disorder, pedophilic disorder, coercive sexual sadism disorder, frotteuristic disorder, paraphilic disorders involving non-consenting individuals, paraphilic disorders involving solitary behavior or consenting individuals (atypical sexual arousal patterns) are all assessed with three items. Two items are related to the given paraphilic disorder-related interest and resulting distress (e.g., “I have had a sustained and intense sexual arousal from the thought or fantasy of exposing my genitals to an unsuspecting individual in public places.”). Participants indicate their level of agreement on a four-point scale (1=“totally disagree”; 4=“totally agree”). One item asks about acting on the given thoughts and fantasies in the past six months (e.g., “I have acted on these thoughts and fantasies in the past six months.”), using a dichotomous answer option (0=“no”; 1=“yes”). Moreover, a fourth item is displayed for paraphilic disorders involving solitary behavior or consenting individuals (atypical sexual arousal patterns), assessing which acts are of interest (i.e., “Which of the following acts are the interest of yours?”, answer options include choking, beating, eating, castration, and free text answer).

##### Sexual desire

The 14-item Sexual Desire Inventory-2 (SDI-2) (Spector, Carey, & Steinberg, 1996) assesses individual (five items, e.g., “How strong is your desire to engage in sexual behavior by yourself?”) and dyadic (nine items, e.g., “How strong is your desire to engage in sexual activity with a partner?”) sexual desire. Participants indicate their answers using eight-point (e.g., 0=“not at all”; 7=“more than once a day”) or nine-point scales (e.g., 0=“no desire”; 8=“strong desire”), with answer options adjusted to items.

##### Sexual function

The five-item Arizona Sexual Experience Scale (ASEX) (McGahuey et al., 2000) assesses the main aspects of sexual function (i.e., sexual drive, arousal, vaginal lubrication/penile erection, ability to reach orgasm, and satisfaction with orgasm) with one item (e.g., “How easily are you sexually aroused (turned on)?”). Participants indicate their answers using a six-point (e.g., 1=“extremely easily”; 6=“never reach orgasm”), with answer options adjusted to each item. A female and a male version of the scale is available with specific male-bodied and female-bodied questions.^4^

##### Sexual assertiveness

The nine-item version of the Sexual Assertiveness Questionnaire (SAQ) (Loshek & Terrell, 2015; Nagy et al., 2021) assesses three dimensions of sexual assertiveness among participants who had sex in the past year: ability to initiate and communicate about sex (three items, e.g., “It is easy for me to discuss sex with my partner.”); ability to refuse unwanted sex (three items, e.g., “It is easy for me to say no if I don’t want to have sex.”); and communication about sexual history and risk (three items, e.g., “I ask my partners about their sexual history.”). Participants indicate their answers on a seven-point scale (1=“strongly disagree”; 7=“strongly agree”).

##### Sexual abuse

The Sexual Abuse History Questionnaire (SAHQ) (Leserman, Drossman, & Li, 1995) assesses participants’ experiencing different types of sexual abuse during their childhood, adolescence, and adulthood, with 12 dichotomous (i.e., “no” and “yes”) questions (e.g., “Has anyone ever forced you to have sex when you did not want this?”).

#### Pornography use-related characteristics

##### General questions about pornography use

Before answering any pornography use-related questions, participants are provided a definition of pornography (Kohut et al., 2019).^5^ Participants report if they have ever used pornography in their life, age at first use, past-year pornography use frequency, time spent with pornography use per each session (in minutes), self-perceived addiction, moral incongruence (Grubbs, Kraus, & Perry, 2019), and past and present treatment-seeking for pornography use (adapted from Kraus, Rosenberg, Martino, Nich, & Potenza, 2017).

##### Pornography use motivations

The 24-item Pornography Use Motivations Scale (PUMS) (Bőthe, Tóth-Király, Bella, et al., 2020) assesses the reasons of pornography use via eight factors: sexual pleasure (three items, e.g., “I watch porn to arouse myself sexually.”), sexual curiosity (three items, e.g., “I watch porn to gather new ideas for sex.”), emotional distraction/suppression (three items, e.g., “I watch porn to suppress my bad mood.”), boredom avoidance (three items, e.g., “I watch porn because I am bored.”), stress reduction (three items, e.g., “I watch porn because it is one of the best ways to relieve stress.”), fantasy (three items, e.g., “I watch porn because I can be a part of things that I cannot experience in real life.”), lack of sexual satisfaction (three items, e.g., “I watch porn because my sexual life is not satisfying.”), and self-exploration (three items, e.g., “I watch porn because I can get to know what I like in sex and what I do not.”). Participants indicate their answers on a seven-point scale (1=“never”; 7=“all the time”).

##### Problematic pornography use

The Problematic Pornography Consumption Scale (PPCS) (Bőthe, Tóth-Király, et al., 2018) and the Brief Pornography Screen (BPS) (Kraus et al., 2020) assess the severity of problematic pornography use in the past six months. The 18-item PPCS includes six factors: salience (three items, e.g., “I felt that porn is an important part of my life.”), tolerance (three items, e.g., “I felt that I had to watch more and more porn for satisfaction.”), mood modification (three items, e.g., “I used porn to restore the tranquility of my feelings.”), conflict (three items, e.g., “Watching porn prevented me from bringing out the best in me.”), withdrawal (three items, e.g., “I became agitated when I was unable to watch porn.”), and relapse (three items, e.g., “I unsuccessfully tried to reduce the amount of porn I watch.”). Participants indicate their answers on a seven-point scale (1=“never”; 7=“all the time”). The BPS is a five-item, one-factor screen (e.g., “You have attempted to “cut back” or stop using porn, but were unsuccessful.”). Participants indicate their answers on a three-point scale (0=“never”; 2=“very often”).

#### Psychological characteristics

##### Religiosity

Three pre-established items (e.g., “I consider myself religious.”) assess participants’ religiosity levels (Grubbs et al., 2019). Participants indicate their answers on a seven-point scale (1=“strongly disagree”; 7=“strongly agree”).

##### Impulsivity

The 20-item Short UPPS-P Impulsive Behavior Scale (UPPS-P) (Billieux et al., 2012) assesses five facets of impulsivity: negative urgency (four items, e.g., “When I am upset I often act without thinking.”), positive urgency (four items, e.g., “When I am really excited, I tend not to think on the consequences of my actions.”), lack of premeditation (four items, e.g., “I usually think carefully before doing anything.”), lack of perseverance (four items, e.g., “I finish what I start.”), and sensation-seeking (four items, e.g., “I sometimes like doing things that are a bit frightening.”). Participants indicate their answers on a four-point scale (1=“I agree strongly”; 4=“I disagree strongly”).

##### Compulsivity

The eight-item Compulsive Personality Assessment Scale (CPAS) (Fineberg, Sharma, Sivakumaran, Sahakian, & Chamberlain, 2007) assesses participants’ compulsive behaviors (e.g., “Are you preoccupied with details, rules, lists, order, organization or schedules to the extent that the major aim of the activity is lost?”). Participants indicate their answers on a five-point scale (0=“not at all characteristic of me”; 4=“entirely characteristic of me”).

##### Basic psychological needs

The 24-item Basic Psychological Needs Satisfaction and Frustration Scale (BPNSFS) (Chen et al., 2015) assesses six dimensions of basic psychological needs: relatedness satisfaction (four items, e.g., “I feel that the people I care about also care about me.”), relatedness frustration (four items, e.g., “I feel excluded from the group I want to belong to.”), competence satisfaction (four items, e.g., “I feel confident that I can do things well.”), competence frustration (four items, e.g., “I have serious doubts about whether I can do things well.”), autonomy satisfaction (four items, e.g., “I feel a sense of choice and freedom in the things I undertake.”), and autonomy frustration (four items, e.g., “I feel forced to do many things I wouldn’t choose to do.”). Participants indicate their answers on a five-point scale (1=“not true at all”; 5=“completely true”).

#### Psychiatric symptomatology and related characteristics

##### Depression and anxiety

The depression and anxiety factors of the short version of the Brief Symptom Inventory (BSI-18) (Asner-Self, Schreiber, & Marotta, 2006) assess participants’ depressive (six items, e.g., “Feeling hopeless about the future”) and anxiety symptoms (six items, e.g., “Nervousness or shakiness inside”) in the past seven days. Participants indicate their answers on a five-point scale (0=“not at all”; 4=“extremely”).

##### Attention deficit hyperactivity disorder

The six-item Adult ADHD Self-Report Scale (ASRS) (Kessler et al., 2005) assesses ADHD symptoms (e.g., “How often do you have difficulty getting things in order when you have to do a task that requires organization?”) in the past six months. Participants indicate their answers on a five-point scale (0=“never”; 4=“very often”).

##### Alcohol use

The 10-item Alcohol Use Disorders Identification Test (AUDIT) (Babor, Higgins-Biddle, Saunders, & Monteiro, 2001) assesses alcohol use-related problems (e.g., “How often during the last year have you failed to do what was normally expected of you because of drinking?”) if participants have used alcohol in the past 12 months. Participants indicate their answers on three-point (e.g., 0=“no”; 4=“yes, during the last year”) and five-point scales (e.g., 0=“never”; 4=“4 or more times a week”), with answer options adjusted to items.

##### Substance use

The 10-item Alcohol, Smoking, and Substance Involvement Screening Test (ASSIST) (Humeniuk et al., 2010) assesses the frequency of participants’ use of different substances (i.e., tobacco products, alcoholic beverages, cannabis, cocaine, amphetamine type stimulants, inhalants, sedatives or sleeping pills, hallucinogens, opioids, and other substances, with free text answers) in the past three months. Participants indicate their answers on a five-point scale (0=“never”; 4=“daily or almost daily”).

##### Gambling

Participants indicate whether they engaged in traditional or online gambling in their lifetime and during the past 30 days. Participants who gambled in the past 30 days also report how many times they engaged in traditional or online gambling during this period.

##### Body Mass Index

Participants’ height (feet, inches or meter, centimeters) and weight (pounds or kilograms) are assessed with two questions.

##### Binge eating

The seven-item Binge Eating Disorder Screener-7 (BEDS-7) (Herman et al., 2016) assesses binge-eating symptoms in participants who report any episodes of excessive overeating in the past three months (e.g., “During your episodes of excessive overeating, how often did you feel disgusted with yourself or guilty afterward?”). Participants indicate their answers on two-point (0=“no”; 1=“yes”) and four-point scales (0=“never or rarely”; 3=“always”), with answer options adjusted to items.

##### Suicidality

The five-item P4 Suicidality Screener (P4) (Dube, Kroenke, Bair, Theobald, & Williams, 2010) assesses participants’ levels of suicidal thoughts and risk among participants who report having suicidal thoughts (e.g., “Have you ever attempted to harm yourself in the past?”). Participants indicate their answers on two-point (0=“no”; 1=“yes”) and three-point scales (0=“not likely at all”; 2=“very likely”), with answer options adjusted to items.

#### Attention testing questions

The survey battery includes three attention-testing questions added to scales that are displayed to all participants (e.g., “We’re evaluating your level of attention, answer “Disagree some” to this question.”). If participants fail at least two out of these questions, their data will be considered invalid (Thomas & Clifford, 2017).

### Statistical analysis

Analyses will be conducted within a structural equation modeling framework. First, we will conduct confirmatory factor analysis to examine the structural validity of each scale. Models will be evaluated based on commonly used goodness-of-fit indices (Browne & Cudeck, 1993; Marsh, Hau, & Grayson, 2005; Schermelleh-Engel, Moosbrugger, & Müller, 2003), such as the Comparative Fit Index or Root-Mean-Square Error of Approximation with its 90% confidence interval. To ensure that comparisons between groups (e.g., genders, countries) are meaningful and to reduce the possibility of measurement biases and invalid comparisons between groups, we will conduct measurement invariance tests (Milfont & Fischer, 2010; Millsap, 2011; Vandenberg & Lance, 2000).

In the next phase, we will conduct latent profile analysis (LPA) to identify individuals’ sexual profiles (Collins & Lanza, 2010). For example, to identify compulsive and non-compulsive sexual behavior profiles, we will use the dimensions of the CSBD-19 as profile indicators, building on the ICD-11-based conceptualization of CSBD (World Health Organization, 2019) and previous LPA studies of CSBD (e.g., Bőthe, Tóth-Király et al., 2020). In another LPA model, we will examine problematic and non-problematic pornography use profiles using the PPCS, BPS, frequency of pornography use, and moral incongruence towards pornography use as profile indicators, building on the engagement and moral incongruence models (Billieux, 2012; Billieux et al., 2019; Grubbs, Perry, et al., 2019; Grubbs & Perry, 2019; Tóth-Király, Bőthe, & Orosz, 2018), and previous empirical findings (Bőthe, Tóth-Király, et al., 2020; Chen, Jiang, Luo, Kraus, & Bőthe, 2021). The selection of the optimal number of classes will be guided by the theoretical meaningfulness and statistical adequacy of the extracted classes (Marsh, Lüdtke, Trautwein, & Morin, 2009), such as the Akaike Information Criterion, entropy, and Lo-Mendell-Rubin Adjusted Likelihood Ratio Test (Lubke & Muthén, 2007). Once the final number of classes are identified, we will compare the identified profiles based on potential predictors (e.g., impulsivity, basic psychological needs) and outcomes (e.g., sexual distress, sexual satisfaction) (Asparouhov & Muthén, 2007), following previous recommendations and best practices (Gillet, Morin, Colombat, Ndiaye, & Fouquereau, 2021; Morin, Meyer, Creusier, & Biétry, 2015). This paper serves as the general description of the ISS study, and additional statistical analyses may be conducted based on the ISS data. Any study based on data from the ISS will have its own preregistration (e.g., including hypothesis and data analysis plan) and will be added as a new component of the study’s OSF project.

### Ethics

The study procedures were conducted following the Declaration of Helsinki. The Institutional Review Board of the Eötvös Loránd University approved the study (2020/474). All participants are informed about the study and provide informed consent.

## Discussion

Following recent calls for integration and rigorous methodological designs in sex research, the ISS was designed and dedicated to understanding a wide range of sexual behaviors, with a focus on CSBD and PPU in diverse populations (Grubbs et al., 2020; Grubbs & Kraus, 2021; Klein et al., 2021). From theoretical and research perspectives, the ISS will provide well-validated, publicly available screening tools (e.g., CSBD-19, PPCS) for researchers, helping to address major issues in the field of potentially compulsive, impulsive, and addictive sexual behaviors research (e.g., difficulties comparing findings across studies given use of different measurements) that may eventually lead to a high-quality, unified assessment (Grubbs et al., 2020). The ISS’ findings may provide important insights to improve the theoretical understanding of CSBD and PPU, and may lay the foundations for improving theoretical models by providing empirical evidence about, for example, potential roles impulsivity and compulsivity may have in CSBD, or moral incongruence may have in PPU (Bőthe, Tóth-Király et al., 2019; Fineberg et al., 2014; Grubbs, Perry, et al., 2019). These insights are important for gaining a deeper understanding of CSBD and PPU (Grubbs et al., 2020). Lastly, following open-science practices to contribute to robust and replicable science, we will preregister all analyses and make open-access study materials. Thus, this study may serve as a blueprint for future large-scale research in addiction and sexuality research.

From an applied perspective, in line with the World Health Organization’s policy and expert recommendations (Chou, Cottler, Khosla, Reed, & Say, 2015; Stein, Szatmari, Gaebel, & Berk, 2020), several sexuality-related measures will be translated and adapted to different languages, and validated in many countries on five continents, including underserved populations and non-WEIRD countries that were underrepresented in previous studies (Grubbs et al., 2020; Klein et al., 2021). These scales will be freely accessible for healthcare professionals (e.g., psychologists), given that maladaptive sexual behaviors may result in severe functional impairment in different life domains (e.g., relationship issues; Kraus et al., 2018). Thus, it is important to assess these behaviors and evaluate the effectiveness of interventions with valid and reliable scales in clinical settings. Results from the current investigation will contribute to the easy identification of different sexual profiles (e.g., high-risk populations) and knowledge about possible antecedents (i.e., risk factors) and outcomes. This may help health care professionals to identify targets of intervention to increase the likelihood of desirable sexual profiles (e.g., higher sexual well-being). On a larger scale, the ISS will introduce potential empirically-supported treatment targets that can be used to improve and develop preventions and interventions.

### Limitations and future studies

Despite the strengths of the ISS, limitations should be considered. The study uses a cross-sectional, self-reported data collection methodology involving self-selected samples; this methodology may be prone to biases (e.g., recall bias). Although the study aims to use large, diverse samples, it will not be representative of all populations in each country, limiting the generalizability of the findings. Future studies will be needed to further examine the validity and reliability of the screening tools planned for use in the ISS in other populations (e.g., adolescents; Bőthe, Vaillancourt-Morel, Dion, Štulhofer, & Bergeron, 2021) and corroborate the identified sexual profiles and profile correlates, with a special focus on non-WEIRD, nationally representative, and treatment-seeking samples (Grubbs et al., 2020; Klein et al., 2021). Moreover, given limits in the survey battery (e.g., relating to respondent burden), it will not be possible to assess all sexuality-related variables (e.g., use of sexual enhancement products; Corazza et al., 2014) or psychosocial characteristics (e.g., sexual self-esteem; Mitchell, Lewis, O’Sullivan, & Fortenberry, 2021) that may be relevant to CSBD, PPU, sexual well-being, or sexual health. Thus, building off ISS findings, future studies will be needed to examine a wider range of variables in relation to CSBD and PPU, preferably in longitudinal settings, providing information about the natural course and temporal stability of assessed and observed associations (Grubbs & Gola, 2019; Grubbs & Kraus, 2021; Štulhofer, Rousseau, & Shekarchi, 2020).

## Conclusions

Addressing the limitations of previous studies (Grubbs et al., 2020; Grubbs & Kraus, 2021; Klein et al., 2021), the ISS is a theory-driven, large-scale, international, multi-lab, multi-language study using cross-sectional survey methods, involving more than 40 countries from five continents. The ISS will provide well-validated, publicly available screening tools, and important insights to improve the theoretical understanding of CSBD and PPU, as well as help to identify empirically supported risk groups and risk and protective factors that may be targeted in future public health campaigns and intervention efforts.

## Funding sources

The research was supported by the Hungarian National Research, Development, and Innovation Office (Grant numbers: KKP126835, K134807). BB was supported by the Merit Scholarship Program for Foreign Students (PBEEE) awarded by the Ministère de l’Éducation et de l’Enseignement Supérieur (MEES) and by a postdoctoral fellowship from the SCOUP Team – Sexuality and Couples – Fonds de recherche du Québec, Société et Culture.

## Author’s contribution

Study concept and design: BB, MK, LN, SWK, MNP, ZD; Obtained funding: BB, ZD; Drafting the article: BB; Revising it critically for important intellectual content: BB, MK, LN, SWK, MNP, ZD; Final approval of the version to be published: BB, MK, LN, SWK, MNP, ZD.

## Conflict of interests

The authors declare no conflict of interest with the content of this manuscript. Dr. Potenza discloses that he has consulted for and advised Game Day Data, Addiction Policy Forum, AXA, Idorsia and Opiant Therapeutics; received research support from the Mohegan Sun Casino, the Connecticut Council on Problem Gambling and the National Center for Responsible Gaming; consulted for or advised legal and gambling entities on issues related to impulse control and addictive behaviors; provided clinical care related to impulse-control and addictive behaviors; performed grant reviews; edited journals/journal sections; given academic lectures in grand rounds, CME events and other clinical/scientific venues; and generated books or chapters for publishers of mental health texts. ELTE Eötvös Loránd University receives funding from the Szerencsejáték Ltd to maintain a telephone helpline service for problematic gambling. ZD has also been involved in research on responsible gambling funded by Szerencsejáték Ltd and the Gambling Supervision Board and provided educational materials for the Szerencsejáték Ltd’s responsible gambling program. The University of Gibraltar receives funding from the Gibraltar Gambling Care Foundation. However, this funding is not related to this study and the funding institution had no role in the study design or the collection, analysis, and interpretation of the data, writing the manuscript, or the decision to submit the paper for publication. ZD is the Editor-in-Chief of the Journal of Behavioral Addictions.

## Footnotes

1 Definition of sexual experiences with a partner used in the study: “Sexual experience with a partner is defined as any activity or behavior (excluding childhood sexual games or possible sexual abuse) that stimulates or arouses a person with the intent to produce an orgasm or sexual pleasure. Think about any kind of sexual experience with a partner.”

2 Before answering any romantic relationship-related questions, participants indicating being in any type of romantic relationship are asked to answer all following questions with respect to their primary partner if they have more than one partner.

3 Definition of sex used for the CSBD-19: “For the purpose of this questionnaire, sex is defined as any activity or behavior that stimulates or arouses a person with the intent to produce an orgasm or sexual pleasure (e.g., self-masturbation or solo sex, using pornography, intercourse with a partner, oral sex, anal sex, etc.). Sexual behaviors may or may not involve a partner.”

4 Participants reporting a different gender identity than their sex assigned at birth, being non-binary, and/or being trans persons are provided a question concerning whether they would like to receive the male-bodied or the female-bodied sexual function scale, or whether they would prefer to skip this entire scale.

5 Definition of pornography used in the study: “Using pornography (porn) means to intentionally look at, read, or listen to: (a) pictures, videos, or films that depict nude individuals or people having sex; or (b) written or audio material that describes nude individuals, or people having sex. Using porn does not involve viewing or interacting with actual, live, nude individuals, or participating in interactive sexual experiences with other human beings in person or online. For example, participating in live sex chat or a camshow, and getting a “lapdance” in a strip club are not considered porn use.”

## References

[B1] Asner-Self, K. K. , Schreiber, J. B. , & Marotta, S. A. (2006). A cross-cultural analysis of the brief symptom inventory-18. *Cultural Diversity & Ethnic Minority Psychology* , 12(2), 367–375. 10.1037/1099-9809.12.2.367. 16719583

[B2] Asparouhov, T. , & Muthén, B. O. (2007). *Wald test of mean equality for potential latent class predictors in mixture modeling* . Technical Report. Muthén & Muthén.

[B3] Babor, T. F. , Higgins-Biddle, J. C. , Saunders, J. B. , & Monteiro, M. G. (2001). *AUDIT: The alcohol use disorders identification test – Guidelines for Use in primary care* (second). World Health Organization.

[B4] Bauer, G. R. , Braimoh, J. , Scheim, A. I. , & Dharma, C. (2017). Transgender-inclusive measures of sex/gender for population surveys: Mixed methods evaluation and recommendations. *Plos One* , 12(5), 1–28. 10.1371/journal.pone.0178043. PMC5444783 28542498

[B5] Beaton, D. E. , Bombardier, C. , Guillemin, F. , & Ferraz, M. B. (2000). Guidelines for the process of cross-cultural adaptation of self-report measures. *Spine* , 25(24), 3186–3191. 11124735 10.1097/00007632-200012150-00014

[B6] Billieux, J. (2012). Problematic use of the mobile phone: A literature review and a pathways model. *Current Psychiatry Reviews* , 8(4), 299–307. 10.2174/157340012803520522.

[B7] Billieux, J. , Flayelle, M. , Rumpf, H.-J. , & Stein, D. J. (2019). High involvement versus pathological involvement in video games: A crucial distinction for ensuring the validity and utility of gaming disorder. *Current Addiction Reports* , 6(3), 323–330. 10.1007/s40429-019-00259-x.

[B8] Billieux, J. , Rochat, L. , Ceschi, G. , Carré, A. , Offerlin-Meyer, I. , Defeldre, A.-C. , … Van der Linden, M. (2012). Validation of a short French version of the UPPS-P impulsive behavior scale. *Comprehensive Psychiatry* , 53(5), 609–615. 10.1016/J.COMPPSYCH.2011.09.001. 22036009

[B20] Bőthe, B. , Vaillancourt-Morel, M.-P. , Dion, J. , Štulhofer, A. , & Bergeron, S. (2021). Validity and reliability of the short version of the problematic pornography consumption scale (PPCS-6-A) in adolescents. *Psychology of Addictive Behaviors* , 35(4), 486–500. 10.1037/adb0000722. 33829815

[B9] Browne, M. W. , & Cudeck, R. (1993). Alternative ways of assessing model fit. *Testing Structural Equation Models* , 21(2), 136–162. 10.1167/iovs.04-1279.

[B10] Bőthe, B. , Bartók, R. , Tóth-Király, I. , Reid, R. C. , Griffiths, M. D. , Demetrovics, Z. , & Orosz, G. (2018). Hypersexuality, gender, and sexual orientation: A large-scale psychometric survey study. *Archives of Sexual Behavior* , 47(8), 2265–2276. 10.1007/s10508-018-1201-z. 29926261

[B11] Bőthe, B. , Koós, M. , Tóth-Király, I. , Orosz, G. , & Demetrovics, Z. (2019). Investigating the associations of adult ADHD symptoms, hypersexuality, and problematic pornography use among men and women on a largescale, non-clinical sample. *Journal of Sexual Medicine* , 16(4), 489–499. 10.1016/J.JSXM.2019.01.312. 30852107

[B12] Bőthe, B. , Lonza, A. , Štulhofer, A. , & Demetrovics, Z. (2020). Symptoms of problematic pornography use in a sample of treatment considering and treatment non-considering men: A network approach. *Journal of Sexual Medicine* , 17(10), 2016–2028. 10.1016/j.jsxm.2020.05.030. 32675049

[B13] Bőthe, B. , Potenza, M. N. , Griffiths, M. D. , Kraus, S. W. , Klein, V. , Fuss, J. , & Demetrovics, Z. (2020). The development of the Compulsive Sexual Behavior Disorder Scale (CSBD-19): An ICD-11 based screening measure across three languages. *Journal of Behavioral Addictions* , 9(2), 247–258. 10.1556/2006.2020.00034. 32609629 PMC8939427

[B14] Bőthe, B. , Tóth-Király, I. , Bella, N. , Potenza, M. N. , Demetrovics, Z. , Orosz, G. , … Orosz, G. (2020). Why do people watch pornography? The motivational basis of pornography use. *Psychology of Addictive Behaviors* , in press. 10.1037/adb0000603. 32730047

[B15] Bőthe, B. , Tóth-Király, I. , Demetrovics, Z. , & Orosz, G. (2021). The short version of the problematic pornography consumption scale (PPCS-6): A reliable and valid measure in general and treatment-seeking populations. *Journal of Sex Research* , 58(3), 342–352. 10.1080/00224499.2020.1716205. 31995398

[B16] Bőthe, B. , Tóth-Király, I. , Griffiths, M. D. , Potenza, M. N. , Orosz, G. , & Demetrovics, Z. (2021). Are sexual functioning problems associated with frequent pornography use and/or problematic pornography use? Results from a large community survey including males and females. *Addictive Behaviors* , 112, 1–9. 10.1016/j.addbeh.2020.106603. 32810799

[B17] Bőthe, B. , Tóth-Király, I. , Potenza, M. N. , Griffiths, M. D. , Orosz, G. , & Demetrovics, Z. (2019). Revisiting the role of impulsivity and compulsivity in problematic sexual behaviors. *Journal of Sex Research* , 56(2), 166–179. 10.1080/00224499.2018.1480744. 29913087

[B18] Bőthe, B. , Tóth-Király, I. , Potenza, M. N. , Orosz, G. , & Demetrovics, Z. (2020). High-frequency pornography use may not always be problematic. *Journal of Sexual Medicine* , 17(4), 793–811. 10.1016/j.jsxm.2020.01.007. 32033863

[B19] Bőthe, B. , Tóth-Király, I. , Zsila, Á. , Griffiths, M. D. , Demetrovics, Z. , & Orosz, G. (2018). The development of the problematic pornography consumption scale (PPCS). *Journal of Sex Research* , 55(3), 395–406. 10.1080/00224499.2017.1291798. 28276929

[B21] Carnes, P. (1983). Out of the shadows: Understanding sexual addiction. CompCare.

[B22] Chen, L. , Jiang, X. , Luo, X. , Kraus, S. W. , & Bőthe, B. (2021). The role of impaired control in screening problematic pornography use: Evidence from cross-sectional and longitudinal studies in a large help-seeking male sample. *Psychology of Addictive Behaviors* . 10.1037/adb0000714. 33764089

[B23] Chen, L. , Luo, X. , Bőthe, B. , Jiang, X. , Demetrovics, Z. , & Potenza, M. N. (2021). Properties of the problematic pornography consumption scale (PPCS-18) in community and subclinical samples in China and Hungary. *Addictive Behaviors* , 112, 106591. 10.1016/j.addbeh.2020.106591. 32768797

[B24] Chen, B. , Vansteenkiste, M. , Beyers, W. , Boone, L. , Deci, E. L. , Van der Kaap-Deeder, J. , … Verstuyf, J. (2015). Basic psychological need satisfaction, need frustration, and need strength across four cultures. *Motivation and Emotion* , 39(2), 216–236. 10.1007/s11031-014-9450-1.

[B25] Chou, D. , Cottler, S. , Khosla, R. , Reed, G. M. , & Say, L. (2015). Sexual health in the international classification of diseases (ICD): Implications for measurement and beyond. *Reproductive Health Matters* , 23(46), 185–192. 10.1016/j.rhm.2015.11.008. 26719010

[B26] Collins, L. M. , & Lanza, S. T. (2010). *Latent class and latent transition analysis: With applications in the social, behavioral, and health sciences* (pp. 1–295). Wiley.

[B27] Collins, L. M. , & Wugalter, S. E. (1992). Latent class models for stage-sequential dynamic latent variables. *Multivariate Behavioral Research* , 27(1), 131–157. 10.1207/s15327906mbr2701_8.

[B28] Corazza, O. , Martinotti, G. , Santacroce, R. , Chillemi, E. , Di Giannantonio, M. , Schifano, F. , & Cellek, S. (2014). Sexual enhancement products for sale online: Raising awareness of the psychoactive effects of yohimbine, maca, horny goat weed, and ginkgo biloba. *BioMed Research International* , 1–13. 10.1155/2014/841798. PMC4082836 25025070

[B29] Derogatis, L. R. , Rosen, R. , Leiblum, S. , Burnett, A. , & Heiman, J. (2002). The Female Sexual Distress Scale (FSDS): Initial validation of a standardized scale for assessment of sexually related personal distress in women. *Journal of Sex & Marital Therapy* , 28(4), 317–330. 10.1080/00926230290001448. 12082670

[B30] Dube, P. , Kroenke, K. , Bair, M. J. , Theobald, D. , & Williams, L. S. (2010). The P4 screener: Evaluation of a brief measure for assessing potential suicide risk in 2 randomized effectiveness trials of primary care and oncology patients. *Primary Care Companion to the Journal of Clinical Psychiatry* , 12(6). 10.4088/PCC.10m00978blu. PMC3067996 21494337

[B31] Fernandez, D. P. , & Griffiths, M. D. (2021). Psychometric instruments for problematic pornography use: A systematic review. *Evaluation* & *the Health Professions* , 44(2), 111–141. 10.1177/0163278719861688. 31284745

[B32] Finch, W. H. , & Bronk, K. C. (2011). Conducting confirmatory latent class analysis using Mplus. *Structural Equation Modeling* , 18(1), 132–151. 10.1080/10705511.2011.532732.

[B33] Fineberg, N. A. , Chamberlain, S. R. , Goudriaan, A. E. , Stein, D. J. , Vanderschuren, L. J. M. J. , Gillan, C. M. , … Potenza, M. N. (2014). New developments in human neurocognition: Clinical, genetic and brain imaging correlates of impulsivity and compulsivity. *CNS Spectrums* , 19(1), 89. 10.1017/S1092852913000801. PMC4113335 24512640

[B34] Fineberg, N. A. , Sharma, P. , Sivakumaran, T. , Sahakian, B. , & Chamberlain, S. (2007). Does obsessive-compulsive personality disorder belong within the obsessive-compulsive spectrum? In *CNS spectrums* (Vol. 12, Issue 6, pp. 467–482). Cambridge University Press. 10.1017/s1092852900015340. 17545957

[B35] Fülöp, F. , Bőthe, B. , Gál, É. , Cachia, J. Y. A. , Demetrovics, Z. , & Orosz, G. (2020). A two-study validation of a single-item measure of relationship satisfaction: RAS-1. *Current Psychology* , 1–10. 10.1007/s12144-020-00727-y.

[B88] Gillet, N. , Morin, A. J. S. , Colombat, P. , Ndiaye, A. , & Fouquereau, E. (2021). Burnout profiles: Dimensionality, replicability, and associations with predictors and outcomes. *Current Psychology* , 2021, 1–19. 10.1007/S12144-021-01807-3.

[B36] Grubbs, J. B. , & Gola, M. (2019). Is pornography use related to erectile functioning? Results from cross-sectional and latent growth curve analyses. *Journal of Sexual Medicine* , 16(1), 111–125. 10.1016/j.jsxm.2018.11.004. 30621919

[B37] Grubbs, J. B. , Hoagland, C. , Lee, B. , Grant, J. , Davison, P. M. , Reid, R. , & Kraus, S. W. (2020). Sexual addiction 25 years on: A systematic and methodological review of empirical literature and an agenda for future research. *Clinical Psychology Review* , 1–15. 10.1016/j.cpr.2020.101925. 33038740

[B38] Grubbs, J. B. , & Kraus, S. W. (2021). Pornography use and psychological science: A call for consideration. *Current Directions in Psychological Science* , 1–8. http://journals.sagepub.com/doi/10.1177/0963721420979594.

[B39] Grubbs, J. B. , Kraus, S. W. , & Perry, S. L. (2019). Self-reported addiction to pornography in a nationally representative sample: The roles of use habits, religiousness, and moral incongruence. *Journal of Behavioral Addictions* , 8(1), 88–93. 10.1556/2006.7.2018.134. 30632378 PMC7044607

[B40] Grubbs, J. B. , & Perry, S. L. (2019). Moral incongruence and pornography use: A critical review and integration. *Journal of Sex Research* , 56(1), 29–37. 10.1080/00224499.2018.1427204. 29412013

[B41] Grubbs, J. B. , Perry, S. L. , Wilt, J. A. , & Reid, R. C. (2019). Pornography problems due to moral incongruence: An integrative model with a systematic review and meta-analysis. *Archives of Sexual Behavior* , 48(2), 397–415. 10.1007/s10508-018-1248-x. 30076491

[B42] Grubbs, J. B. , Volk, F. , Exline, J. J. , & Pargament, K. I. (2015). Internet pornography use: Perceived addiction, psychological distress, and the validation of a brief measure. *Journal of Sex & Marital Therapy* , 41(1), 83–106. 10.1080/0092623X.2013.842192. 24341869

[B43] Gudicha, D. W. , Tekle, F. B. , & Vermunt, J. K. (2016). Power and sample size computation for Wald tests in latent class models. *Journal of Classification* , 33(1), 30–51. 10.1007/s00357-016-9199-1.

[B44] Herman, B. K. , Deal, L. S. , Dibenedetti, D. B. , Nelson, L. , Fehnel, S. E. , & Michelle Brown, T. (2016). Development of the 7-Item Binge-Eating disorder screener (BEDS-7). *Primary Care Companion to the Journal of Clinical Psychiatry* , 18(2). 10.4088/PCC.15m01896. PMC4956427 27486542

[B45] Humeniuk, R. , Henry-Edwards, S. , Ali, R. , Poznyak, V. , Monteiro, M. G. , & World Health Organization . (2010). *The alcohol, smoking and substance involvement screening test (ASSIST)_ manual for use in primary care* . World Health Organization. www.who.int/substance_abuse.

[B46] Kafka, M. P. (2010). Hypersexual disorder: A proposed diagnosis for DSM-V. *Archives of Sexual Behavior* , 39(2), 377–400. 10.1007/s10508-009-9574-7. 19937105

[B47] Kessler, R. C. , Adler, L. , Ames, M. , Demler, O. , Faraone, S. , Hiripi, E. , … Walters, E. E. (2005). The World health organization adult ADHD self-report scale (ASRS): A short screening scale for use in the general population. *Psychological Medicine* , 35(2), 245–256. 10.1017/S0033291704002892. 15841682

[B48] Klein, V. , Savaş, Ö. , & Conley, T. D. (2021). How WEIRD and androcentric is sex research? Global inequities in study populations. *The Journal of Sex Research* , 1–8. 10.1080/00224499.2021.1918050. 33939579

[B49] Kohut, T. , Balzarini, R. N. , Fisher, W. A. , Grubbs, J. B. , Campbell, L. , & Prause, N. (2019). Surveying pornography use: A shaky science resting on poor measurement foundations. *Journal of Sex Research* , 1–21. 10.1080/00224499.2019.1695244. 31821049

[B50] Koós, M. , Bőthe, B. , Orosz, G. , Potenza, M. N. , Reid, R. C. , & Demetrovics, Z. (2021). The negative consequences of hypersexuality: Revisiting the factor structure of the Hypersexual Behavior Consequences Scale and its correlates in a large, non-clinical sample. *Addictive Behaviors Reports* , 13, 100321. 10.1016/j.abrep.2020.100321. 33364331 PMC7750154

[B51] Koós, M. , Fuss, J. , Klein, V. , Nagy, L. , Kraus, S. W. , Potenza, M. N. , … Bőthe, B. (2021). The development of the Paraphilic Disorders Scale (PDS) . In preparation.

[B52] Kor, A. , Fogel, Y. A. , Reid, R. C. , & Potenza, M. N. (2013). Should hypersexual disorder be classified as an addiction? *Sexual Addiction and Compulsivity* , 20(1–2), 27–47. 10.1080/10720162.2013.768132. PMC3836191 24273404

[B53] Kowalewska, E. , Gola, M. , Kraus, S. W. , & Lew-starowicz, M. (2020). Spotlight on compulsive sexual behavior disorder: A systematic review of research on women. *Neuropsychiatric Disease and Treatment* , 16, 2025–2043. 10.2147/NDT.S221540. 32943868 PMC7478918

[B54] Kraus, S. W. , Gola, M. , Grubbs, J. B. , Kowalewska, E. , Hoff, R. A. , Lew-Starowicz, M. , … Potenza, M. N. (2020). Validation of a brief pornography screen across multiple samples. *Journal of Behavioral Addictions* , 9(2), 259–271. 10.1556/2006.2020.00038. 32644937 PMC8939429

[B55] Kraus, S. W. , Krueger, R. B. , Briken, P. , First, M. B. , Stein, D. J. , Kaplan, M. S. , … Reed, G. M. (2018). Compulsive sexual behaviour disorder in the ICD-11. *World Psychiatry* , 17(1), 109–110. 10.1002/wps.20499. 29352554 PMC5775124

[B56] Kraus, S. W. , Martino, S. , & Potenza, M. N. (2016). Clinical characteristics of men interested in seeking treatment for use of pornography. *Journal of Behavioral Addictions* , 5(2), 169–178. 10.1556/2006.5.2016.036. 27348557 PMC5387768

[B57] Kraus, S. W. , Rosenberg, H. , Martino, S. , Nich, C. , & Potenza, M. N. (2017). The development and initial evaluation of the pornography-use avoidance self-efficacy scale. *Journal of Behavioral Addictions* , 6(3), 354–363. 10.1556/2006.6.2017.057. 28889754 PMC5700731

[B58] Kraus, S. W. , Voon, V. , & Potenza, M. N. (2016a). Additional challenges and issues in classifying compulsive sexual behavior as an addiction. *Journal of Sex Research* , 111(9), 181–198. 10.1111/add.13297.

[B59] Kraus, S. W. , Voon, V. , & Potenza, M. N. (2016b). Should compulsive sexual behavior be considered an addiction? *Addiction* , 111(12), 2097–2106. 10.1111/add.13297. 26893127 PMC4990495

[B60] Lawrance, K. , & Byers, E. S. (1998). Interpersonal exchange model of sexual satisfaction questionnaire. In C. M. Davis , W. L. Yarber , R. Baureman , G. Schreer , & S. L. Davis (Eds.), *Sexuality related measures: A compendium* (second, pp. 514–519). Gage.

[B61] Leserman, J. , Drossman, D. A. , & Li, Z. (1995). The reliability and validity of a sexual and physical abuse history questionnaire in female patients with gastrointestinal disorders. *Behavioral Medicine* , 21(3), 141–150. 10.1080/08964289.1995.9933752. 8789650

[B62] Loshek, E. , & Terrell, H. K. (2015). The development of the sexual assertiveness questionnaire (SAQ): A comprehensive measure of sexual assertiveness for women. *The Journal of Sex Research* , 52(9), 1017–1027. 10.1080/00224499.2014.944970. 25211014

[B63] Lubke, G. , & Muthén, B. O. (2007). Performance of factor mixture models as a function of model size, covariate effects, and class-specific parameters. *Structural Equation Modeling: A Multidisciplinary Journal* , 14(1), 26–47. 10.1080/10705510709336735.

[B64] Marsh, H. W. , Hau, K.-T. , & Grayson, D. (2005). Goodness of fit in structural equation models. In A. Maydeu-Olivares & J. J. McArdle (Eds.), *Contemporary psychometrics: A festschrift for Roderick P. McDonald* (pp. 275–340). Lawrence Erlbaum Associates Publishers.

[B65] Marsh, H. W. , Lüdtke, O. , Trautwein, U. , & Morin, A. J. S. S. (2009). Classical latent profile analysis of academic self-concept dimensions: Synergy of person- and variable-centered approaches to theoretical models of self-concept. *Structural Equation Modeling* , 16(2), 191–225. 10.1080/10705510902751010.

[B66] McGahuey, C. A. , Gelenberg, A. J. , Laukes, C. A. , Moreno, F. A. , Delgado, P. L. , McKnight, K. M. , & Manber, R. (2000). The Arizona sexual experience scale (Asex): Reliability and validity. *Journal of Sex & Marital Therapy* , 26(1), 25–38. 10.1080/009262300278623. 10693114

[B67] Milfont, T. L. , & Fischer, R. (2010). Testing measurement invariance across groups: Applications in cross- cultural research. Probando la invariancia de mediciones entre varios grupos: Aplicaciones en la investigación transcultural. *International Journal of Psychological Research* , 3(31), 2011–2079. 10.1007/s11135-007-9143-x.

[B68] Millsap, P. (2011). *Statistical approaches to measurement invariance* . New York, NY: Taylor & Francis.

[B69] Mitchell, K. R. , Lewis, R. , O’Sullivan, L. F. , & Fortenberry, J. D. (2021). What is sexual wellbeing and why does it matter for public health? *Lancet Public Health* , 1–6. 10.1016/S2468-2667(21)00099-2. PMC7616985 34166629

[B89] Morin, A. J. S. , Meyer, J. P. , Creusier, J. , & Biétry, F. (2015). Multiple-group analysis of similarity in latent profile solutions. *Organizational Research Methods* , 19(2), 231–254. 10.1177/1094428115621148.

[B70] Nagy, L. , Koós, M. , Kraus, S. W. , Potenza, M. N. , Demetrovics, Z. , & Bőthe, B. (2021). The short version of the Sexual Assertiveness Questionnaire (SAQ) . In preparation.

[B71] Pâquet, M. , Rosen, N. O. , Steben, M. , Mayrand, M. H. , Santerre-Baillargeon, M. , & Bergeron, S. (2018). Daily anxiety and depressive symptoms in Couples coping with Vulvodynia: Associations with women’s pain, women’s sexual function, and both partners’ sexual distress. *Journal of Pain* , 19(5), 552–561. 10.1016/j.jpain.2017.12.264. 29309891

[B72] Potenza, M. N. , Gola, M. , Voon, V. , Kor, A. , & Kraus, S. W. (2017). Is excessive sexual behaviour an addictive disorder? *Lancet Psychiatry* , 4(9), 663–664. 10.1016/S2215-0366(17)30316-4. 28853408

[B73] Prause, N. , Janssen, E. , Georgiadis, J. , Finn, P. , & Pfaus, J. (2017). Data do not support sex as addictive. *The Lancet Psychiatry* , 4(12), 899. 10.1016/S2215-0366(17)30441-8. 29179928

[B74] Reid, R. C. , Carpenter, B. N. , Hook, J. N. , Garos, S. , Manning, J. C. , Gilliland, R. , … Fong, T. (2012). Report of findings in a DSM-5 field trial for hypersexual disorder. *Journal of Sexual Medicine* , 9(11), 2868–2877. 10.1111/j.1743-6109.2012.02936.x. 23035810

[B75] Sassover, E. , & Weinstein, A. (2020). Should compulsive sexual behavior (CSB) be considered as a behavioral addiction? A debate paper presenting the opposing view. *Journal of Behavioral Addictions* , 1–14. 10.1556/2006.2020.00055. 32997646 PMC9295215

[B76] Schermelleh-Engel, K. , Moosbrugger, H. , & Müller, H. (2003). Evaluating the fit of structural equation models: Tests of significance and descriptive goodness-of-fit measures. *MPR-Online* , 8(2), 23–74.

[B77] Slavin, M. N. , Blycker, G. R. , Potenza, M. N. , Bőthe, B. , Demetrovics, Z. , & Kraus, S. W. (2020). Gender-related differences in associations between sexual abuse and hypersexuality. *Journal of Sexual Medicine* , 17(10), 2029–2038. 10.1016/j.jsxm.2020.07.008. 32792283 PMC7875089

[B78] Spector, I. P. , Carey, M. P. , & Steinberg, L. (1996). The sexual desire inventory: Development, factor structure, and evidence of reliability. *Journal of Sex & Marital Therapy* , 22(3), 175–190. 10.1080/00926239608414655. 8880651

[B79] Stein, D. J. , Szatmari, P. , Gaebel, W. , & Berk, M. (2020). Mental, behavioral and neurodevelopmental disorders in the ICD-11: An international perspective on key changes and controversies. *Journal Fur Neurologie, Neurochirurgie Und Psychiatrie* , 21(1), 30–32. 10.1186/s12916-020-1495-2. PMC6983973 31983345

[B80] Štulhofer, A. , Rousseau, A. , & Shekarchi, R. (2020). A two-wave assessment of the structure and stability of self-reported problematic pornography use among male Croatian adolescents. *International Journal of Sexual Health* , 32(2), 151–164. 10.1080/19317611.2020.1765940.

[B81] Thomas, K. A. , & Clifford, S. (2017). Validity and Mechanical Turk: An assessment of exclusion methods and interactive experiments. *Computers in Human Behavior* , 77, 184–197.

[B82] Tóth-Király, I. , Bőthe, B. , & Orosz, G. (2018). Seeing the forest through different trees: A social psychological perspective of work addiction. *Journal of Behavioral Addictions* , 7(4), 875–879. 10.1556/2006.7.2018.122. 30556783 PMC6376368

[B83] Turchik, J. A. , & Garske, J. P. (2009). Measurement of sexual risk taking among college students. *Archives of Sexual Behavior* , 38(6), 936–948. 10.1007/s10508-008-9388-z. 18563548

[B84] Vandenberg, R. J. , & Lance, C. E. (2000). A review and synthesis of the measurement invariance literature: Suggestions, practices, and recommendations for organizational research. *Organizational Research Methods* , 3(1), 4–70. 10.1177/109442810031002.

[B85] Weinrich, J. D. (2014). On the design, development, and testing of sexual identity questions: A discussion and analysis of Kristen miller and J. Michael Ryan’s work for the national health interview survey. *Journal of Bisexuality* , 14, 502–523. 10.1080/15299716.2014.952052.

[B86] Wordecha, M. , Wilk, M. , Kowalewska, E. , Skorko, M. , Lapinski, A. , & Gola, M. (2018). “Pornographic binges” as a key characteristic of males seeking treatment for compulsive sexual behaviors: Qualitative and quantitative 10-week-long diary assessment. *Journal of Behavioral Addictions* , 7(2), 433–444. 10.1556/2006.7.2018.33. 29865868 PMC6174597

[B87] World Health Organization . (2019). International statistical classification of diseases and related health problems (11th ed.). https://icd.who.int/. 10.2471/BLT.25.294650 PMC13531092 42683092

